# Reply to: No specific relationship between hypnotic suggestibility and the rubber hand illusion

**DOI:** 10.1038/s41467-022-28178-y

**Published:** 2022-01-28

**Authors:** P. Lush, A. K. Seth

**Affiliations:** 1 Sackler Centre for Consciousness Science, University of Sussex, Falmer, BN1 9RH UK; 2 Department of Informatics, Chichester Building, University of Sussex, Falmer, BN1 9RH UK; 3 Canadian Institute for Advanced Research, Program on Brain, Mind, and Consciousness, 661 University Avenue, Toronto, ON M5G 1M1 Canada

**Keywords:** Psychology, Human behaviour

**replying to** Ehrsson et al. *Nature Communications* 10.1038/s41467-022-28177-z (2022)

We welcome the discussion generated by our study^1^ examining the relationship between trait response to imaginative suggestion (phenomenological control)^2^ and measures of the rubber hand illusion (RHI) and mirror synaesthesia. Ehrsson and colleagues focus on the RHI and claim that our results are consistent with RHI effects being driven primarily by multisensory mechanisms. We disagree. Our results show that RHI reports are, at least partially, likely to be driven by top-down phenomenological control in response to demand characteristics (“the totality of cues which convey an experimental hypothesis to the subject”^3^). Ehrsson et al. provide a number of re-analyses of our data to support their argument. However, all but one confirm the findings we presented in the target paper, and the sole new analysis is insensitive and therefore uninformative. The disagreement is therefore not about data or analyses, but interpretation. It is important to note also that, in our view, Ehrsson et al.’s commentary fails to appreciate the implications of a critical issue: the asynchronous condition offers no protection against demand characteristic effects (including faking, imagination and phenomenological control)^4^.

There are two main points of disagreement. The first regards our reported null relationship between hypnotisability (phenomenological control in a ‘hypnotic’ context) and a difference measure of subjective report (the mean agreement score for three statements describing either referred touch or ‘ownership’ experience; the difference measure is the difference in mean agreement between synchronous and asynchronous conditions). Ehrsson et al. argue that this result contradicts our claims. They extend our control group analysis which showed this null relationship^1^ to the whole sample and replicate our reported null result. Contrary to their argument, this new analysis is consistent with our results and interpretation (they also extend our control group analysis of proprioceptive drift and hypnotisability to the whole sample; however, the data are insensitive and no conclusions follow^5^). Critically, Ehrsson et al. do not acknowledge that their interpretation of the difference between the synchronous condition and an asynchronous control condition is confounded by demand characteristics. For a control condition to be valid, all factors except the manipulated factor (in this case the timing of multisensory stimuli) must be held constant across conditions. However, expectancies are not matched across these conditions. As we reported in the original article^1^ and has since been shown elsewhere^4,6,7^, participant expectancies are greater for the synchronous than asynchronous condition.

Indeed, analysis of the expectancy data from the target article (*n* = 353)^1^ shows hypnotisability does not predict the difference in expectancies between synchronous and asynchronous conditions:, *b* = −0.16 Likert units subjective response per SWASH unit, *SE* = 0.09, *t* = 1.78, *P* = 0.072, B_H(0,0.25)_ = 0.07 (B based on the SWASH/report correlation). *r*_s_ = −0.08, 95% CI [−0.18, 0.03]. Participant expectancies arising from demand characteristics readily account for our reported null result, since these expectancies do not vary with the level of hypnotisability. Our interpretation is that the invariant difference in expectancies across participants can be met either by generating experience, or by other demand characteristic effects (note, however, that differences in reported experience can also arise from differences in suggestion difficulty^4^). In other words, participants can respond to the differing demand characteristics by either generating the corresponding experiences (if they have high trait capacity for phenomenological control, i.e. hypnotisability) or by response bias (if they have low capacity for phenomenological control). This applies equally to implicit measures of the RHI (e.g., skin conductance response and proprioceptive drift), as we have shown by measuring expectancies for these measures; as with subjective report, people expect the patterns of results that are typically obtained in RHI experiments^7^.

We turn now to the second point of disagreement: whether our reported relationships between hypnotisability and the RHI are substantial enough to pose a threat to existing multisensory theories of the RHI. Ehrsson et al. do not take into account our linear models of raw effects but instead interpret standardised correlation coefficients, arguing that the effect size, *R*^2^ = 0.09, is weak. We disagree. Cohen describes an *R*^2^ of 0.09 as a medium-size correlation, visible to the naked eye^8^. Funder & Ozer argue that, in psychology, an *R*^2^ of 0.09 indicates “a large effect that is potentially powerful in both the short and the long run”^9^. That aside, we do not interpret standardised correlation coefficients in the target paper because linear models of raw effects are far more informative. The linear model in the target article shows a 0.6 increase in mean score for the three illusion statements (7-point scale) for each 1 point increase in SWASH (5-point scale). In our view, this is not a weak relationship (see 'Scatter plots showing linear regression (*n*  =  353 participants) of synchronous condition rubber hand illusion measures on hypnotisability and expectancies' in the target paper^1^).

One might further worry, as Reader, Trifonova and Ehrsson^10^ (see also ref. ^11^) point out, that mean illusion report measures are problematic, because referred touch response is generally greater than ‘ownership’ response, and the two varieties of experience may dissociate. In our data (*n* = 353), mean agreement (maximum of 3) for the statements describing an experience of referred touch was 1.9, *SE* = 0.1 (S1) and 1.2, *SE* = 0.1 (S2). For the crucial statement describing ‘ownership’ experience (S3), mean agreement was 0.7, *SE* = 1.0. To investigate the relationship between hypnotisability and ‘ownership’ specifically, we conducted a linear regression (Fig. 1). Minimum ‘ownership’ agreement (a score of 1) is predicted only for SWASH scores greater than two (the top 31% of hypnotisability scores)—underlining that on average it is participants in the higher ranges of trait phenomenological control who report experiencing ownership in the RHI^11^. It is also worth noting that the ownership report is confounded by order effects. In a re-analysis of data from the target paper, mean agreement with the ownership statement is seen only in the group who underwent the asynchronous ‘control’ task first and who were therefore exposed to (and hence aware of) all measurement procedures (e.g., text describing illusion experience and accompanying report scales) before participating in the synchronous condition^12^.

**Fig. 1:**
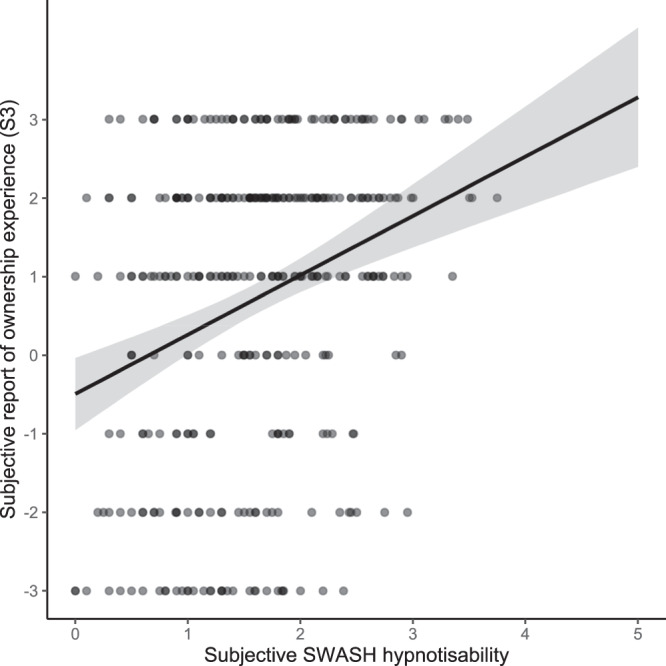
Agreement with the statement “I felt as if the rubber hand were my hand” (S3; ‘ownership’) on hypnotisability. *b* = 0.76 Likert units subjective response per SWASH unit, *SE* = 0.13, *t* = 5.79, *P* < 0.001, 95% CI [0.50, 1.01] B_H(0,1.4)_ = 4.2 × 10^6^ (B calculated as in Lush et al^1^.). *r*_s_ = 0.27, 95% CI [0.17, 0.36]. Source data are provided as a Source Data file.

Ehrsson et al. also draw attention to residual levels of RHI report at low levels of hypnotisability by analysing arbitrarily split quartile groups. In our view their analysis is redundant; the linear model in the target article clearly shows these residual effects^1^. Our interpretation is that these residual effects may reflect other effects arising from demand characteristics (e.g., bias or compliance effects)^2,11^. Note that, for the crucial ownership statement, there may not be a residual effect (Fig. 1).

Ehrsson et al. criticise our literature review. We point out that the most common method in the 20 most influential RHI papers is to test the difference between synchronous and asynchronous conditions, and then interpret only the synchronous condition^1^ (the approach employed in their commentary). However, they assert (without evidence) that difference measures are more widely used for interpretation in less influential papers. Even if this were to be so, it would not undermine our claims because, as we have explained, difference measures are confounded by expectancies^4,6,7^. Furthermore, even if these papers happened to be the only papers which employed the methods discussed, given how influential they are, the implications for the understanding of rubber hand effects would still be great. We disagree with the claim that (by focusing on the synchronous condition for exploratory regression analyses) we ignored the control measures, which we in fact describe in detail in the target paper^1^ and elsewhere^2,4,7,11–13^.

All further issues raised in the Matters Arising are addressed briefly here, or in the original paper, or in our earlier preprint^13^.

Demand characteristics may also account for the results of the more fine-grained (2AFC; two-alternative forced choice) approaches described by Ehrsson et al. There is no reason why these measures should be any more resistant to demand characteristic effects than the more common methods addressed in Lush et al.^1^.

A simple explanation for the similar correlations between RHI reports and phenomenological control for both synchronous and asynchronous conditions is that both reflect suggestion effects. Lower mean scores for visual hallucination than, e.g., ownership may reflect the lower expectancies for visual hallucination experience^4,6,7^ or the relatively high difficulty of generating visual hallucination experience^4,13^.

Ehrsson et al. refer to our declared deviation from pre-registration for correlational analyses and question the value of our exploratory analyses. We could not proceed with preregistered investigation of expectancy-related differences between instruction conditions because instructions did not change expectancies^1^. It transpired that the implicit demand characteristics inherent in the RHI could not be overcome by our instructions^1,13^. This, along with all other points raised regarding pre-registered analyses, is stated in the target paper^1^. We note that Ehrsson et al.’s arguments are also not based on preregistered analyses and are therefore also exploratory and post-hoc. Any general concerns about the interpretation of non-preregistered work apply equally to their own conclusions here and elsewhere.

The RHI is just one of several examples of relationships between phenomenological control and experimental measures described in the target paper^1^ and elsewhere^14^. It just happens to be among the first examples investigated regarding the hypothesis that expectancies can drive experience in psychology studies, just as they do in direct imaginative suggestion. The key issue at stake is that failing to control demand characteristics can result in incorrect inference regarding mechanisms^7^. Other RHI measures may also be confounded (e.g., cross-modal congruency or 2AFC tasks) but this has not been tested^1,4,7,11,13^. Future experiments, with adequate control conditions, are needed to establish whether mechanisms beyond demand characteristic effects (e.g., multisensory integration) play a significant role in RHI measures. Beyond addressing potential confounds, such experiments may open fresh opportunities for psychological research by shedding new light on the complex interactions between bottom-up and top-down influences that shape all perceptual experiences, within and beyond the laboratory.

## Reporting summary

Further information on research design is available in the Nature Research Reporting Summary linked to this article.

## Supplementary information

Reporting summary

## Data Availability

All analyses presented in this manuscript are re-analyses of data from a previous publication^1^. The data that support the findings reported in this manuscript are available at https://osf.io/kx6hw/. There are no restrictions on data availability. Source data are provided with this paper.

## Source data

Source Data

## Code availability

A JASP file that allows the reproduction of the linear regression presented in Fig. 1 is available at https://osf.io/kx6hw/. Source data are provided with this paper.

## References

[1] Lush P (2020). Trait phenomenological control predicts experience of mirror synaesthesia and the rubber hand illusion. Nat. Commun..

[2] Dienes, Z. et al. Phenomenological control as cold control. *Psychol.**Conscious. Theory Res. Pract*. 10.1037/cns0000230 (2020).

[3] Orne MT (1962). On the social psychology of the psychological experiment: with particular reference to demand characteristics and their implications. Am. Psychol..

[4] Lush P (2020). Demand characteristics confound the rubber hand illusion. Collabra Psychol..

[5] Dienes, Z. Using Bayes to get the most out of non-significant results. *Front. Psychol*. **5**, 781 (2014). 10.3389/fpsyg.2014.00781 PMC4114196 25120503

[6] Reader, A. T. What do participants expect to experience in the rubber hand illusion? 10.31234/osf.io/d8x9y (2021).

[7] Lush P, Seth AK, Dienes Z (2021). Hypothesis awareness confounds asynchronous control conditions in indirect measures of the rubber hand illusion. R. Soc. open Sci..

[8] Cohen, J. *Statistical Power Analysis for the Behavioral Sciences* (Academic Press, 2013).

[9] Funder DC, Ozer DJ (2019). Evaluating effect size in psychological research: sense and nonsense. Adv. Methods Pract. Psychol. Sci..

[10] Reader AT, Trifonova VS, Ehrsson HH (2021). The relationship between referral of touch and the feeling of ownership in the rubber hand illusion. Front. Psychol..

[11] Roseboom, W. & Lush, P. Serious problems with interpreting rubber hand illusion experiments. 10.31234/osf.io/uhdzs (2020).

[12] Lush, P. Order effects in the rubber hand illusion. 10.31234/osf.io/amsrp (2021).

[13] Seth, A., Roseboom, W., Dienes, Z. & Lush, P. What’s up with the Rubber Hand Illusion? 10.31234/osf.io/b4qcy (2021).

[14] Lush, P., Dienes, Z., Seth, A. & Scott, R. B. Trait phenomenological control predicts visually evoked auditory response. 10.31234/osf.io/x9eud (2021).

